# AAV-NDI1 Therapy Provides Significant Benefit to Murine and Cellular Models of Glaucoma

**DOI:** 10.3390/ijms25168876

**Published:** 2024-08-15

**Authors:** Sophia Millington-Ward, Arpad Palfi, Ciara Shortall, Laura K. Finnegan, Ethan Bargroff, Iris J. M. Post, John Maguire, Mustapha Irnaten, Colm O′Brien, Paul F. Kenna, Naomi Chadderton, G. Jane Farrar

**Affiliations:** 1 The School of Genetics and Microbiology, Smurfit Institute of Genetics, Trinity College Dublin, Dublin 2, D02VF25 Dublin, Ireland; palfia@tcd.ie (A.P.); shortac@tcd.ie (C.S.); lafinneg@tcd.ie (L.K.F.); bargrofe@tcd.ie (E.B.); ipost@tcd.ie (I.J.M.P.); pfkenna@tcd.ie (P.F.K.); gjfarrar@tcd.ie (G.J.F.); 2 The Research Foundation, Royal Victoria Eye and Ear Hospital, Dublin 2, D02XK51 Dublin, Ireland; john.maguire@olchc.ie; 3 UCD Clinical Research Centre, Mater Misericordiae University Hospital, Dublin 7, D07K201 Dublin, Ireland; mustapha.irnaten2@ucd.ie (M.I.); cobrien@mater.ie (C.O.)

**Keywords:** glaucoma, DBA/2J mouse, mitochondria, ROS, gene therapy, AAV, retina, NDI1, lamina cribrosa

## Abstract

Glaucoma, a leading cause of blindness, is a multifactorial condition that leads to progressive loss of retinal ganglion cells (RGCs) and vision. Therapeutic interventions based on reducing ocular hypertension are not always successful. Emerging features of glaucoma include mitochondrial dysfunction and oxidative stress. In the current study, NDI1-based gene therapy, which improves mitochondrial function and reduces reactive oxygen species, was delivered intraocularly via an adeno-associated viral vector (AAV). This AAV-NDI1 therapy protected RGCs from cell death in treated (1552.4 ± 994.0 RGCs/mm^2^) versus control eyes (1184.4 ± 978.4 RGCs/mm^2^, *p* < 0.05) in aged DBA/2J mice, a murine model of glaucoma. The photonegative responses (PhNRs) of RGCs were also improved in treated (6.4 ± 3.3 µV) versus control eyes (5.0 ± 3.1 µV, *p* < 0.05) in these mice. AAV-NDI1 also provided benefits in glaucomatous human lamina cribrosa (LC) cells by significantly increasing basal and maximal oxygen consumption rates and ATP production in these cells. Similarly, NDI1 therapy significantly protected H_2_O_2_-insulted primary porcine LC cells from oxidative stress. This study highlights the potential utility of NDI1 therapies and the benefits of improving mitochondrial function in the treatment of glaucoma.

## 1. Introduction

Glaucoma is a complex group of optic neuropathies and a leading cause of blindness. It is thought to affect in the region of 70–80 million people worldwide, with approximately 10% of patients being blind. Indeed, 2.93% of people in Europe aged between 40 and 80 years have been reported to have glaucoma, rising to 10% of persons over 90 years of age [1,2,3,4]. The condition is classified into open-angle glaucoma, where the anterior chamber remains open, and closed-angle glaucoma, with closure of the drainage angle leading to impairment of the aqueous outflow. It is a multifactorial condition with risk factors, including advanced age, genetics, myopia, and elevated intraocular pressure (IOP), with the greatest being the latter [1]. At present, the only modifiable risk factor is IOP; therefore, current glaucoma treatments are based on lowering IOP with topical eye drops, surgery, or laser therapy. However, the outcomes of these treatments are variable, with some patients not responding and/or suffering from serious side effects, while others present with a normotensive form of glaucoma, where increased IOP is absent [5]. Notably, all glaucoma patients suffer a characteristic progressive loss of the visual field, mainly due to the progressive loss of retinal ganglion cells (RGCs). Pathology occurs in the RGC soma in the inner retina and in RGC axons, which radiate toward and constitute the main component of the optic nerve head (ONH) and optic nerve, which conveys visual information to the brain [6].

The exact mechanism by which RGCs are lost in glaucoma is not yet fully understood, nor is the mechanism by which increased IOP may lead to their death. However, mechanical distortions of the ONH due to increased IOP are thought to lead to remodelling of the extracellular matrix and fibrosis in the LC [7]. This, in turn, may lead to ischaemia-hypoxia damage, blockage of axonal transport, and deprivation of neurotrophic factors, as well as functional changes in astrocytes and microglia, all of which may contribute to RGC death [8,9,10,11,12]. RGCs have high energy demands, making them highly reliant on healthy mitochondrial function. Mitochondrial dysfunction resulting in reduced mitochondrial bioenergetics and increased oxidative stress has been observed in RGCs of glaucoma patients and multiple animal models of glaucoma and is known to contribute to the apoptosis of RGCs [13,14,15,16,17,18,19,20,21,22,23,24].

Oxidative stress and mitochondrial dysfunction have been associated with many other neurodegenerative conditions, including Parkinson’s disease, Alzheimer’s disease, motor neuron disease, and retinal degeneration, such as age-related macular degeneration (AMD) and diabetic retinopathy [25,26,27,28]. In glaucoma, oxidative stress and mitochondrial dysfunction have been reported not only in RGCs [13,14,15,16,17,18,19,20,21,22,23,24,29] but also in the trabecular meshwork (TM) [30,31,32,33,34,35,36,37,38,39,40],the LC [41] and ONH [42,43,44,45,46,47,48]. The TM is a key regulator of IOP and aqueous drainage in the anterior chamber, while the LC is a sieve-like structure at the ONH, through which the RGC axons and retinal blood vessels pass.

Glaucoma is a complex disease with diverse phenotypes; hence, generating appropriate animal and cellular models for this condition is challenging. In vivo models are frequently based on surgically induced increases in IOP [49]. Genetic models of glaucoma offer a more gradual onset and progression and, therefore, may be more representative of the condition. The DBA/2J mouse model [22,50] displays some phenotypic similarities with glaucoma. For example, mice have excessive iris pigment dispersion, which blocks the TM, leading to aqueous humour accumulation, a steady increase in IOP between 6 and 11 months of age, and loss of RGCs [51,52]. Reduced ATP in RGCs is thought to interfere with the transmission of the visual signal along the optic nerve [53,54], which precedes axon structural changes and impairments in anterograde transport [55,56]. Electrophysiological (ERG) decline [57,58], morphological changes in mitochondria [59], and inflammation in RGC compartments [60,61,62,63] have also been demonstrated in DBA/2J mice. 

LC cells from glaucoma patients have been used as an in vitro model of the disease and shown to have reduced levels of ATP, as well as reduced oxidative phosphorylation and elevated glycolysis [64]. An elevated number of mitochondria and increased calcium and ROS levels suggest mitochondrial dysfunction [41,65,66]. LC cells experimentally exposed to H_2_O_2_ to mimic the oxidative stress environment associated with glaucoma represent another in vitro model for this disease. 

NDI1 is a nuclear-encoded complex I equivalent derived from *S. cerevisiae*. A variety of optimised NDI1 constructs have been shown by us to provide benefits in in vitro and in vivo models of dry AMD, primary fibroblasts from a Leber hereditary optic neuropathy (LHON) patient, and a rotenone-induced murine model of RGC dysfunction [67,68,69,70]. In the current study, an enhanced NDI1 gene (eNdi1) was explored as a gene-independent gene therapy for glaucoma [70]. The therapy is aimed at improving mitochondrial function and decreasing ROS in a variety of glaucoma models: aged DBA/2J mice, primary human glaucomatous LC cells, and primary porcine LC cells insulted with H_2_O_2_.

## 2. Results

It is well established that mitochondrial dysfunction and increased ROS levels are key features of many neurodegenerative disorders, including glaucoma. In the current study, the benefit of eNdi1 constructs delivered via adeno-associated viral vectors (AAV-eNdi1) driven by *CMV* and *CAG* promoters was analysed in two in vitro models of glaucoma and in vivo in the DBA/2J murine model of glaucoma. Effects on mitochondrial function and disease progression have been analysed.

### 2.1. AAV-eNdi1 Provides Benefit in H_2_O_2_-Insulted Porcine LC Cells

**Figure 1:**
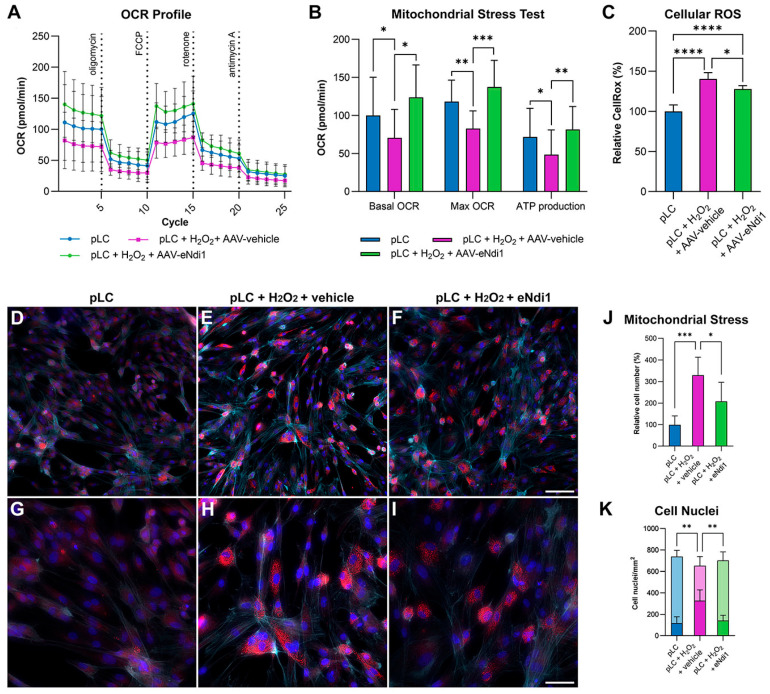
AAV-eNdi1 protects pLC cells from H_2_O_2_-insult. (**A**,**B**) 3 × 10^4^ pLC cells were seeded into XFe96 Seahorse plates. The following day, cells were transduced with AAV-eNdi1 (MOI = 8 × 10^3^). 48 h post-transduction, transduced (*n* = 4–5 wells), and untransduced pLC cells (n > 10 wells) were insulted with 750 μM H_2_O_2_ for 1 h and subsequently underwent a mitochondrial stress test. The mitochondrial stress test was also performed on pLC cells that had not been insulted (n > 10 wells). Notably, H_2_O_2_ insult significantly reduced basal and maximal oxygen consumption rates (OCRs) and ATP production compared to control pLC cells. However, basal and maximal OCRs and ATP production were rescued in AAV-eNdi1-treated cells that received the insult. Statistics were performed on 7–8 replicate mitochondrial stress test assays. (**C**) 4 × 10^4^ pLC cells were seeded in 48-well plates. The following day, cells were transduced with AAV-eNdi1 (*n* = 6, MOI = 2.5 × 10^3^). 24 h post-seeding transduced cells and an additional *n* = 6 wells with untransduced pLC cells were insulted with 750 μM H_2_O_2_ for 30 min, after which a ROS (CellROX) assay was performed. Control pLC cells were also assayed (*n* = 8). The Y-axis represents the median CellROX fluorescence, a marker for ROS, and the levels relative to control pLC cells. (**D–K**) 2 × 10^6^ pLC cells were transfected with 2 mg eNdi1 plasmid (**C**,**D**,**G**,**H**) or a control plasmid (AAV-MCS; **E**,**F**,**I**,**J**). Cells were insulted 48 h post-transfection with 750 μM H_2_O_2_ for 1 h, fixed, and stained with Phalloidin-iFluor 647 (F-actin, light blue), anti-CPN60 (mitochondrial stress marker, red) antibody, and DAPI (nuclear stain, dark blue). eNdi1-treated and H_2_O_2_-insulted cells (**F**,**I**; *n* = 6) were compared to plasmid AAV-MSC-treated (vehicle control) and H_2_O_2_-insulted cells (**E**,**H**; *n* = 6) and control pLC cells that had not been insulted (**D**,**G**; *n* = 5). (**J**) The relative number of CPN60-positive cells with elevated fluorescence intensity was determined. (**K**) Cell nuclei/mm^2^ with high DAPI fluorescence intensity indicating dense nuclei (dark blue, pink, and green) and all cell nuclei/mm^2^ with DAPI fluorescence (pale blue, pink, and green) were quantified. Scale bars: 50 μm (**F**) and 100 μm (**I**). * *p* < 0.05; ** *p* < 0.01; *** *p* < 0.001; **** *p* < 0.0001 (ANOVA). Error bars represent SD values. In (**K**), ** refers to the difference between the numbers of dense nuclei.

To determine whether AAV-eNdi1 could rescue cell models of glaucoma, H_2_O_2_-insulted primary porcine LC cells (pLC cells) were utilised. pLC cells were transduced with AAV-eNdi1 (MOI = 8 × 10^3^). 48 h post-transduction, cells were insulted with 750 μM H_2_O_2_ for 1 h and compared in a mitochondrial stress test to uninsulted control cells and insulted cells that had not received AAV-eNdi1. The basal oxygen consumption rate (OCR, a measure of mitochondrial function) of control pLC cells was considered to be 100%. H_2_O_2_ insult significantly reduced basal OCR levels (70.6 ± 37.3% versus 100.0 ± 50.2%), maximal OCR levels (82.7 ± 23.3% versus 118.2 ± 28.3%), and ATP production (48.6 ± 32.4% versus 71.8 ± 37.7%), indicating that oxidative stress significantly reduces mitochondrial function in H_2_O_2_-insulted pLC cells. Notably, the basal OCR, maximal OCR, and ATP production levels were significantly higher in H_2_O_2_-insulted cells that had received AAV-eNdi1 (123.9 ± 39.4%, 137.5 ± 32.3%, and 81.5 ± 28.1%, respectively), indicating rescue of mitochondrial function in the model (Figure 1A,B).

Elevated ROS can be a hallmark feature of mitochondrial dysfunction and a symptom of glaucoma [71], and the ability of AAV-eNdi1 to reduce ROS levels in the H_2_O_2_-insulted pLC model was investigated. Cells were transduced with AAV-eNdi1 (MOI = 2.5 × 10^3^). 24 h post-transduction, cells were insulted with 750 μM H_2_O_2_ for 30 min (*n* = 6), and ROS levels compared to levels in untransduced insulted cells (*n* = 6) and control cells that had not been transduced or insulted (*n* = 8). Median CellROX fluorescence levels, indicating relative ROS levels, in control cells were considered to be 100%. Relative ROS levels were significantly higher in H_2_O_2_-insulted cells than in control cells (140.5 ± 7.91% versus 100.0 ± 8.05%, respectively); however, these levels were reduced in H_2_O_2_-insulted cells that had received AAV-eNdi1 (127.9 ± 4.1%; Figure 1C).

The effects of eNdi1-treatment in the H_2_O_2_-insulted pLC model were also analysed by immunocytochemistry. Cells were transfected with eNdi1 (*n* = 6; Figure 1F,I) or pAAV-MCS (*n* = 6; Figure 1E,H) plasmid DNA; 48 h later, cells were insulted with 750 μM H_2_O_2_ for 1 h, and then fixed. Transfected cells were compared to control pLC cells that had not been transfected or insulted (*n* = 5; Figure 1D,G). Cells were stained with Phalloidin-iFluor 647 (detecting F-actin) and anti-CPN60 antibody (mitochondrial stress marker, Cy3-labelled secondary antibody), and nuclei were counterstained with DAPI. H_2_O_2_ insult resulted in a change in the morphology of the pLC cells, as visualised by F-actin staining (Figure 1D–I). The insulted cells were more spindle-shaped compared to control cells, which displayed more cell spreading, almost filling up the available surface area. The pattern of CPN60 staining was more punctuated in H_2_O_2_-insulted cells, while the CPN60 staining intensity was also significantly increased compared to uninsulted control cells (Figure 1 D–I). The relative number of cells with elevated intensity of the CPN60 label was quantified by fluorescence microscopy (Figure 1J) and compared to insulted cells that had been treated with eNdi1; the relative number of untreated control cells was taken to be 100% (± 41.8%). The percentage of cells with elevated levels of CPN60 in H_2_O_2_-insulted cells with eNdi1 or without eNdi1 was 205.9 ± 88.2% and 329.4 ± 82.4%, respectively, indicating that eNdi1 significantly reduced mitochondrial stress in insulted cells (*p* < 0.05). H_2_O_2_ insult also resulted in an increased number of apoptotic cell nuclei (identified by DAPI staining; Figure 1D–I), which were also quantified by fluorescence microscopy (Figure 1K). In particular, apoptotic nuclei were 142 ± 49/mm^2^ and 326.5 ± 100.2/mm^2^ (*p* < 0.01) for eNdi1 treated and untreated H_2_O_2_-insulted cells, respectively, while they were 117.9 ± 58.5/mm^2^ in untreated control cells. The total number of nuclei was 703.5 ± 79.8/mm^2^, 654.3 ± 85.2/mm^2,^ and 739.7 ± 57.5/mm^2^, respectively, in the above groups of cells and did not differ significantly. As such, eNdi1 treatment provided benefits in three phenotypes; eNdi1 treated cells maintained their spread-out morphology, mitochondrial stress was significantly reduced, and there were fewer apoptotic cells, the latter two quantified by microscopy (Figure 1C–K).

In summary, in the H_2_O_2_-insulted pLC model, eNdi1 rescued mitochondrial function, reduced cellular ROS levels, and reduced mitochondrial stress and apoptosis.

### 2.2. AAV-eNdi1 Provides Benefit in Human Glaucomatous LC Cells

**Figure 2:**
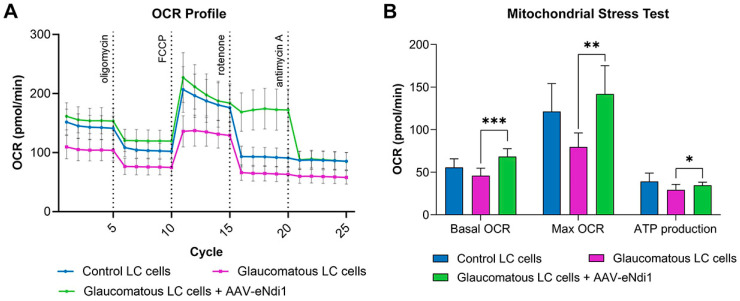
AAV-eNdi1 rescues mitochondrial function in glaucomatous human lamina cribrosa (LC) cells. 1.5 × 10^4^ human glaucomatous and control primary LC cells were seeded into XFe96 Seahorse plates. The following day, >5 wells were transduced with AAV-eNdi1 (MOI = 8 × 10^5^). (**A**) 48 h post-transduction, transduced glaucomatous cells (*n* > 5 wells), untransduced control (*n* > 10 wells), and glaucomatous patient cells (*n* > 10 wells) underwent a mitochondrial stress test. (**B**) Basal and maximal oxygen consumption rates (OCRs) and ATP production were significantly increased in treated glaucomatous cells. Notably, statistics were performed on eight replicate mitochondrial stress test assays performed on a single patient and a control LC line. * *p* < 0.05, ** *p* < 0.01 *** *p* < 0.001. Control and glaucomatous LC cells were compared using paired *t*-tests. The parameters of treated and untreated glaucomatous and control cells were compared using ANOVA with Šídák’s multiple comparison test. Error bars represent SD values.

Mitochondrial function was analysed in a second in vitro model for glaucoma in human primary glaucomatous LC cells (Figure 2). LC cells from an unaffected, age-matched person were used as a control. Glaucomatous LC cells were transduced with AAV-eNdi1 (MOI = 8 × 10^5^), and mitochondrial function was assessed 48 h later using a mitochondrial stress test. Treatment of glaucomatous LC cells with AAV-eNdi1 significantly increased the basal oxygen consumption rate (OCR; 136.70 ± 18.63 pmol/min versus 91.90 ± 17.87 pmol/min; *p* < 0.001) and maximal OCR (284.00 ± 66.47 pmol/min versus 159.30 ± 33.19 pmol/min; *p* < 0.01), and there was a trend toward increased ATP production levels (69.20 ± 7.35 pmol/min versus 58.55 ± 12.92 pmol/min; *p* < 0.05). Notably, OCR levels remain high in AAV-eNdi1-treated cells post rotenone insult because, in contrast to endogenous complex I, eNdi1 is insensitive to rotenone, and hence respiration continues in the rotenone-insulted cells (A).

### 2.3. AAV-eNdi1 Provides Benefit in the DBA/2J Murine Model of Glaucoma

**Figure 3:**
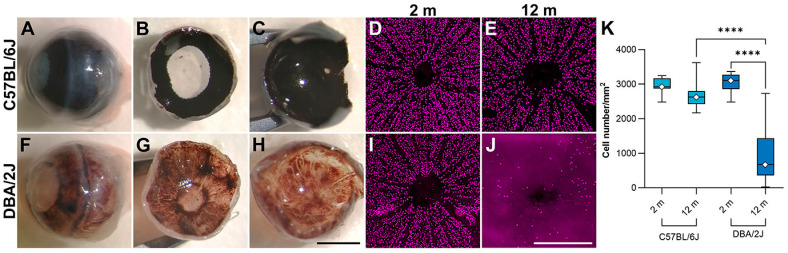
Pigmentary dispersal and quantification of retinal ganglion cells in the DBA/2J eye. Eyes from two- and twelve-month-old (2 m and 12 m) DBA/2J and C57BL/6J (wt) mice were enucleated and fixed in 4% paraformaldehyde. The eyes were dissected at the ora serrata, and the lens and retina were removed. Aberrant pigmentary dispersal in the whole eye (**A**,**F**), iris (**B**,**G**), and RPE (**C**,**H**) of DBA/2J versus C57BL/6J (wt) mouse eyes was observed at 12 m. Retina wholemounts from 2 m and 12 m mice were stained with an anti-BRN3A primary antibody (detected with a Cy3-conjugated secondary antibody). The wholemounts were analysed via fluorescence microscopy, and RGC numbers were automatically quantified (*n* = 9–14; **K**). No significant difference was found between 2 m C57BL/6J (**D**,**K**; *n* = 9), 12 m C57BL/6J (**E**,**K**; *n* = 14), and 2 m DBA/2J (**I**,**K**; *n* = 14) mice. However, 12 m DBA/2J mice (**J**,**K**; *n* = 7) had significantly fewer RGCs. Scale bars: 2 mm (**H**) and 0.5 mm (**J**). **** *p* < 0.0001. RGC numbers were compared using ANOVA with Šídák’s multiple comparisons test. Error bars represent SD values.

In addition to the in vitro models of glaucoma, H_2_O_2_-insulted pLC cells, and human glaucomatous LC cells, the murine DBA/2J mouse model of glaucoma was utilised in the study. This model is late-onset with a variable phenotype; hence, it was necessary to use a large number of mice. Consistent with the literature, pigmentary displacement in the iris was observed in twelve-month-old (aged) DBA/2J mice (Figure 3A–H). In addition, a significant loss of RGCs was observed in aged DBA/2J mice (984.7 *±* 867.3; *n* = 7) compared to two-month-old DBA/2J mice (3050.2 *±* 301.3; *n* = 14) two-month-old C57BL/6J mice (2972.4 ± 234.4 RGCs/mm; *n* = 9) and twelve-month-old C57BL/6J mice (2647.3 *±* 353.9 RGCs/mm; *n* = 14). Notably, differences in RGC numbers of two-month DBA/2J, two-month C57BL/6J, and twelve-month-old C57BL/6J mice were not significant (Figure 3D,E,I–K).

**Figure 4:**
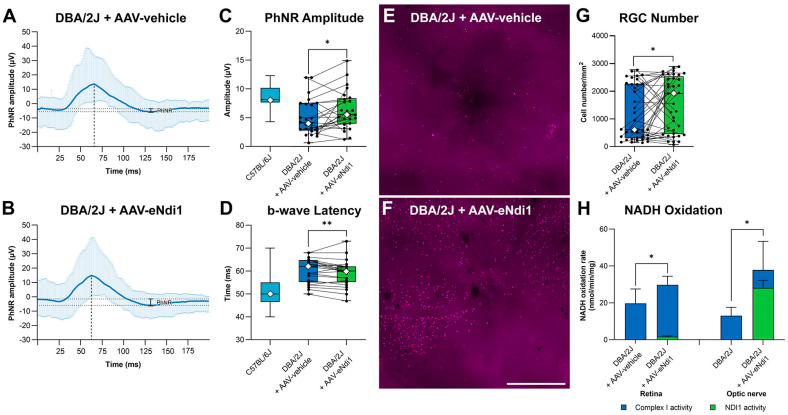
AAV-eNdi1 preserves visual function and RGC number and increases NADH oxidation rates in DBA/2J mice. (**A**,**B**) Photopic single-flash cone (SFC) responses. To establish the functional effects of AAV-eNdi1 in vivo, 2-month-old DBA/2J mice were intravitreally injected with 4.1 × 10^7^ vg AAV-eNdi1 in one eye (green), while the contralateral eye received an equal volume of vehicle control (blue). Ten months post-treatment, the amplitude of the photonegative response (PhNR; indicated between the two horizontal dotted lines) of photopic single-flash cone (SFC) responses were measured, and readings from untreated control eyes (**A**) compared to treated contralateral eyes (**B**). The latency of the b-wave (ms), indicated by the vertical dotted line, was also determined. The SDs of the SFC responses are represented in pale blue. (**C**) The bar chart represents individual PhNR amplitudes established per eye (*n* = 28) and PhNR b-wave latency (**D**), and wild-type C57BL/6J values (turquoise) are also provided. (**C**,**D**) Eyes were subsequently enucleated and fixed in 4% pfa, and wholemounts prepared (*n* = 37). (**E**,**F**) Wholemount retinas were stained with BRN3A immunocytochemistry. (**G**) Retinal ganglion cells (RGCs) were automatically quantified. (**H**) NADH oxidation rate assay was performed on retinas (*n* = 7) and optic nerves (*n* = 4 per group, each sample comprising four pooled optic nerves). Scale bar (**F**): 500 µm. * *p* < 0.05, ** *p* < 0.01, Student’s paired *t*-test except for comparisons between C57BL/6J and DBA/2J data (**C**,**D**), where Student’s *t*-tests were used. Error bars represent SD values.

Evaluation of RGC loss in AAV-eNdi1 treated mice was undertaken as a measure of therapeutic efficacy. Two-month-old DBA/2J mice were intravitreally injected with 4.1 × 10^7^ vg AAV-eNdi1 in one eye, while the contralateral eye received an equal volume of PBS. Ten months post-injection, photonegative responses (PhNRs), representing RGC function, were analysed from photopic single-flash cone responses and compared to PhNRs of age-matched C57BL/6J control mice (Figure 4A–C). AAV-eNdi1-treated eyes displayed significantly improved PhNR infections compared to control PBS-injected eyes (6.4 ± 3.3 µV and 5.0 ± 3.1 µV, respectively, *p* < 0.05, paired *t*-test; *n* = 25). Notably, when the untreated eyes were assigned a value of 100% to normalise for the variable nature of the DBA/2J murine model, PhNRs in treated versus untreated eyes, were 127.0 ± 66.9% and 100 ± 62.1%, respectively (paired Student’s *t*-test; *p* < 0.05). Importantly, PhNRs in treated DBA/2J mouse eyes were not significantly different from PhNRs in control C57BL/6J mice (8.6 ± 2.2 µV, *n* = 9, *p* = 0.07, Student’s *t*-test).

In addition, the implicit time of the single-flash cone b-waves was found to be significantly delayed in untreated DBA/2J versus control C57BL/6J mice (Figure 4A,B,D; 60.1 ± 5.2 ms, *n* = 32, and 51.2 ± 6.6 ms, *n* = 28, respectively, *p* < 0.05, Student’s *t*-test). Notably, this delay was significantly reduced in AAV-eNdi1-treated DBA/2J mice versus untreated DBA/2J mice (58.8 ± 5.5 ms and 60.1 ± 5.2 ms, respectively, *n* = 28, *p* < 0.01, paired *t*-test), although this did not reach levels found in C57BL/6J mice. Implicit times of photopic a-waves did not differ between the treated and untreated DBA/2J mouse eyes.

To determine whether the functional benefits mediated by AAV-eNdi1 in DBA/2J mice (Figure 4A–D) were reflected at the histological level and resulted in RGC preservation, retinal wholemounts were prepared to enable RGC analysis. RGCs were labelled with BRN3A immunocytochemistry and quantified via fluorescence microscopy at twelve-month of age (10 months post-AAV delivery). Representative wholemounts of control PBS-injected and AAV-eNdi1 treated retinas are shown in (Figure 4E,F). RGC numbers in wholemounts were compared in AAV-eNdi1-treated (1552.4 ± 994.0 RGCs/mm^2^) versus untreated (1184.4 ± 978.4) eyes were significantly higher (*n* = 37, *p* < 0.05, paired *t*-test; Figure 4G).

Complex I and eNdi1 catalyse the oxidation of NADH; hence, the measurement of the NADH oxidation rate in isolated retinal mitochondria enables direct evaluation of complex I and eNdi1 function. By including rotenone in the NADH oxidation rate assay, the NADH oxidation rate arising from eNdi1 activity, which is rotenone-insensitive, can be distinguished from the NADH oxidation rate arising from endogenous complex I activity (which is sensitive to rotenone; Figure 4H). NADH oxidation rate was determined by measuring the rate of decrease in absorbance at 340 nm, which occurs when NADH is oxidised to NAD+ and normalised to total protein [72]. The NADH oxidation activities in mitochondria extracted from treated and untreated DBA/2J retinas were 29.8 ± 4.7 nmol/min/mg and 19.8 ± 7.8 nmol/min/mg, respectively (*n* = 7, *p* < 0.05), and in optic nerves were 37.9 ± 15.5 nmol/min/mg and 13.0 +/− 4.5 nmol/min/mg, respectively (*n* = 4 per group, each sample comprising four pooled optic nerves, *p* < 0.05). These data indicate that AAV-eNdi1 significantly increases NADH oxidation activity in both the whole retina and optic nerve. This increase is more pronounced in the optic nerve, as post-intravitreal injection AAV-eNdi1 transduces RGCs and the optic nerve efficiently. Hence, in mitochondria from the whole retina, where RGCs are thought to represent only 0.5–1.3% of retinal cells, depending on the mouse strain and age, the overall effect is less pronounced [73].

In conclusion, these data indicate that AAV-eNdi1 provides a functional benefit in DBA/2J mice with an increase in PhNRs and a reduction in the latency of the single-flash b-wave observed. In addition, RGCs were protected, and NADH oxidation rates increased in treated versus untreated DBA/2J mice.

## 3. Discussion

Glaucoma is a complex and common condition with diverse genetic and phenotypic profiles. Currently, there are thought to be around 170 genetic loci associated with an increased risk of developing primary open-angle glaucoma (POAG) [74,75,76]. In addition, environmental factors such as diet, corticosteroid use, and diabetes mellitus are some risk factors identified to date [77]. Given the complexity of the condition, overarching next-generation therapies aimed at preserving RGCs or lowering IOP via the TM and ciliary body are being considered, with multiple IOP-lowering drugs in use. Potential novel therapies under investigation include those targeting genes involved in decreasing aqueous humour production and increasing its outflow [78] and protective agents, such as growth factors and immunomodulators. Additionally, because apoptosis is considered to be an ultimate driver of RGC cell death in glaucoma [79], anti-apoptotic agents for glaucoma are also being investigated. 

For example, AAV-delivered matrix metalloproteinase-3 (*MMP-3*)-mediated gene therapy has been shown to increase outflow and reduce IOP in a variety of glaucoma models [80]. In addition, sirtuin 1 (*SIRT-1*), which encodes an NAD+-dependent deacetylase, has been shown to protect RGCs in an optic nerve crush [81,82] and an IOP model of glaucoma [83]. Anti-apoptotic agents such as *Xiap* have been shown to provide neuroprotection in a number of rodent models [84,85]. Similarly, *SIRT-6* overexpression has been shown to be protective in glaucoma models [86], as has Tau protein modulation [87], among others. Oral administration of nicotinamide (vitamin B_3_), an adenine dinucleotide (NAD+) precursor, as well as AAV-delivered murine nicotinamide nucleotide adenylyltransferase 1 (*Nmnat1*), which catalyses the synthesis of NAD, have been shown to protect RGCs in the DBA/2J mouse [14]. Typically, these new innovative therapies are still in the early stages of development. However, multiple clinical trials for glaucoma are in progress to analyse the effects of orally delivered nicotinamide [88,89,90]. Due to the patient burden and some nicotinamide-induced liver damage observed during a recent trial [91], gene therapies, such as a therapy expressing, for example, human *NMNAT1* (see above), may be a preferable option.

Clearly, many factors may contribute to the development of glaucoma, including environmental and genetic factors. However, while the exact mechanism remains unknown, mitochondrial dysfunction and oxidative stress are typical hallmarks of glaucoma [92,93]. Hence, in the current study, eNdi1 gene therapy aimed at directly improving mitochondrial function, reducing ROS, and preserving RGCs was investigated. eNdi1 gene therapy has previously been shown to boost mitochondrial function and reduce oxidative stress in a range of murine and cellular models of dry AMD and chemically induced models of RGC loss [67,68,70]. Generally, a limitation of gene therapy studies on glaucoma has been that no model recapitulates all aspects of the human condition. While non-human primate (NHP) models of glaucoma, such as the NHP experimental glaucoma (EG) model and laser-induced models, have a similar retinal architecture to the human retina, these models also have limitations, such as late and variable age of onset and phenotypic variability, as well as high cost and ethical considerations [94,95]. To mitigate against this limitation, a variety of different models, all displaying aspects of the condition, were chosen for the current study. Firstly, aged DBA/2J mice, a high-tension model, were utilised. Williams et al. demonstrated mitochondrial abnormalities as an early driver of glaucoma in DBA/2J mice. Genes involved in mitochondrial dysfunction and oxidative phosphorylation pathways were significantly enriched in RGCs from the mice. Indeed, important metabolites that are key to mitochondrial function and protection from ROS, including NAD+ and NADH, were significantly decreased from 4 months of age [14]. Interestingly, mitochondrial dysfunction was observed before degeneration was apparent. It is believed that in this model, elevated IOP leads to an increase in dephosphorylation of mitochondrial dynamin-related protein 1 (Drp1), a fundamental component of mitochondrial fission, leading to an increase in mitochondrial fragmentation and apoptosis of neuronal cells [96,97]. In addition, an increase in apolipoprotein A–I binding protein (AIBP, an important regulator of autophagy) expression has also been observed, resulting in increased mitochondrial fission and reduced ATP production [98]. Similar changes have been observed in multiple other models of glaucoma [99,100,101]. In addition, primary glaucomatous LC cells, a patient-derived model, were used in the current study. These cells have been shown to display reduced mitochondrial function and increased oxidative stress, known markers of glaucoma [64,65,66]. Finally, primary pLC cells insulted with H_2_O_2_ were utilised. H_2_O_2_ has been shown to induce the expression of profibrotic extracellular matrix genes in LC and TM cells [102,103,104], similar to that observed in glaucoma patients, as well as increase ROS [105,106].

Notably, mitochondrial function was increased, and ROS was reduced in cell models, while the PhNR responses and RGC cell numbers were increased in DBA/2J mice, demonstrating functional and histological rescue by the therapy. Interestingly, the implicit time of the light-adapted single-flash cone b-wave was found to be delayed in aged DBA/2J mice, a finding also reported by Harazny et al. [58], who also found an extremely degraded flicker electroretinography (ERG) response in these mice. The flicker response is known to be driven by the inner retinal cone system, specifically ON and OFF bipolar cells with initial input from cone photoreceptor cells, while the single-flash light-adapted b-wave is resoundingly ON and OFF bipolar cells in origin. AAV-eNdi1 was shown to reduce the time delay of the single-flash light-adapted b-wave in aged DBA/2J mice, indicating that the observed benefit may originate directly from some degree of protection of second-order neurones, or, more probably indirectly, as a result of RGC preservation. Analyses in DBA/2J mice were undertaken at 10 months post-injection. We and others have previously shown that AAV2/2 transduces mainly RGCs following intravitreal injection, and the area of transduction encompasses most of the retina [69,107]. In addition, the expression is known to be maintained for at least 18 months [108]. 

In conclusion, in the current study, we have shown that eNdi1 directly increases mitochondrial function in cell models of glaucoma. Additionally, we showed that ROS and mitochondrial stress were reduced, while cellular health was improved. RGCs and visual function were increased in the DBA/2J model of glaucoma, a mouse model known to display mitochondrial dysfunction, indicating that directly increasing mitochondrial function can protect mice and provide significant benefits. Of note, a key differentiator between other approaches, such as *NMNAT1* and *SIRT-1* gene therapies, which also modulate mitochondrial dysfunction, and the therapeutic strategy for glaucoma demonstrated in the current study, is that eNdi1, as a complex I equivalent, directly participates in the electron transport chain. Given the increasing prevalence of glaucoma in the ageing population, it is imperative that novel therapies, such as the current once-off gene therapy, are further developed and commercialised.

## 4. Materials and Methods

### 4.1. Study Design

An enhanced NDI1 gene, eNdi1, was delivered via a recombinant AAV or a plasmid to models of glaucoma: DBA/2J mice, LC cells from a glaucoma patient, and porcine LC cells that had been insulted with H_2_O_2_. Functional benefit was demonstrated using ERG, and histological benefit was demonstrated by assessing the preservation of RGCs. In addition, increased NADH oxidation rates, reduced cellular ROS, improved OCRs (indicating mitochondrial function), and improved morphological features were assessed.

### 4.2. Plasmid Constructs and AAV Production 

**Figure 5:**
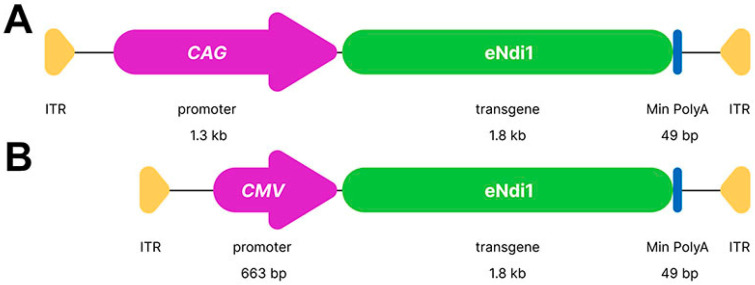
(**A**,**B**) Diagrammatic representation of AAV-eNdi1. Inverted terminal repeats (ITRs), *CAG* (**A**) and *CMV* (**B**) promoters, eNdi1, and minimal PolyAs are indicated with the sizes provided.

Plasmid constructs eNdi1 (patent no 10220102) and *CAG*-*EGFP* have been described previously [109]. *CMV* and *CAG* promoter-driven eNdi1 (Figure 5) and *CAG*-*EGFP* were packaged into AAV2/2 by helper virus-free triple transfection as previously described [110], generating AAV-eNdi1 and AAV-EGFP. Genomic titres (vg/ml) were determined by RT-qPCR [111].

### 4.3. LC Cell Characterisation, Cell Culture and Mitochondrial Stress Test

Eyes from a male donor diagnosed with glaucoma (age 86) and a normal female control (age 88) were obtained from the Central Florida (Tampa) Eye Bank for Transplant and Research within 24 h post-mortem. The presence of glaucoma was confirmed based on retrieved medical records and/or confirmation of previous glaucoma diagnosis by family members. The eyes were obtained and managed in compliance with the Declaration of Helsinki for research involving human tissues. The eyes were cut flush with the posterior sclera and imaged from the posterior cut surface. The optic nerves of the specimens were examined, and the specimens were further classified. LC cells were isolated from single ONH explants from human donor eyes as previously described [112]. Cells were characterised for markers including α-SMA stain (expressed by LC cells), GFAP (astrocyte marker), and Iba1 (microglial marker) as previously described [113,114]. The cells were kindly provided by Professor Abbot Clark (Alcon Labs, Fort Worth, TX and Duke University, Durham, NC, USA). Mitochondrial stress tests were performed on a Seahorse XFe96 analyser (Agilent) as follows. 1.5 × 10^4^ human LC cells were seeded into wells of a 96-well Seahorse plate (Agilent). Two days post-seeding, cells were transduced with *CAG*-driven AAV-eNdi1, MOI = 8 × 10^5^. Two days post-transduction, a mitochondrial stress test was performed (Agilent Technologies, Santa Clara, CA, USA). Injection cycles were 5× for basal OCR, 5× following oligomycin (1.0 μM), 5× following FCCP (1.0 μM), 5× following rotenone (0.5 μM), and 5× following antimycin A (0.5 μM) injections [115,116]. Groups consisted of a minimum of 5 replicate wells, and mitochondrial stress tests were performed 8 times.

Porcine LC cells were isolated from fresh eyes obtained from a local abattoir, as previously described [112]. LC cells (3 × 10^4^ LC cells were seeded into the wells of a 96-well Seahorse plate (Agilent Technologies, Santa Clara. CA, USA). Two days post-seeding, the cells were transduced with *CAG*-driven AAV-eNdi1, MOI = 8 × 10^3^. Two days post-transduction, cells were insulted with 750 μM H_2_O_2_ in a medium at 37 °C for 1 h. Subsequent to insult, cells underwent an Agilent mitochondrial stress test as described above. Groups consisted of a minimum of 5 replicate wells, and mitochondrial stress tests were performed 7 times.

### 4.4. Cellular ROS Assay

5 × 10^4^ cells primary porcine LC cells were seeded into 96-well culture plates (Sarstedt, Nümbrecht, Germany) and were transduced the following day with *CAG*-driven AAV-eNdi1 (MOI = 2.5 × 10^3^). Two days post-transduction, cells were insulted with 750 μM H_2_O_2_ in a medium for 30 min at 37 °C. Subsequent to insult, a cellular ROS assay was performed using CellROX green (Thermo Fisher, Waltham, MA, USA) and flow cytometry as previously described [67].

### 4.5. pLC Transfection Immunocytochemistry

2 × 10^6^ pLC cells were transfected (Amaxa biosystems, Nucleofector II/2B) with the Cell Line Nucleofector Kit L, using a 4.5:1 ratio of supplement to Nucleofector solution, and program C-005 with 2µg of AAV-eNdi1 plasmid DNA [70], or pAAV-MCS (Agilent Technologies Inc, Santa Clara, USA; control) as per manufacturer’s instructions. (Lonza Group AG, Basel, Switzerland). Following transfection, 1.5 × 10^5^ LC cells were seeded onto 8-well imaging slides (Milenyi Biotec, Bergisch Gladback, Germany). 48 h post-transfection cells were insulted with 750 μM H_2_O_2_ for 1 h at 37 °C, then gently washed in PBS and fixed in 4% paraformaldehyde in PBS at RT for 20 min. Cells were stained for F-actin (Phalloidin-iFluor 647, Abcam, Cambridge, UK) and CPN60 (EnCor Biotechnology, Gainesville, FL, USA), and nuclei were counterstained with DAPI as previously described [67].

### 4.6. Intravitreal Injection and Electroretinography in Mice

DBA/2J and C57BL/6J mice (Jackson Laboratories, Bar Harbor, ME, USA) were maintained under specific pathogen-free conditions. The injections were performed in strict compliance with the European Union Regulations 2012 (S.I. no. 543 of 2021) and the Association for Research in Vision and Ophthalmology (ARVO) statement for the use of animals. The study was approved by the local Animal Research Ethics Committee of Trinity College Dublin (Ref. no. 140514/240320). Intravitreal injections were performed as previously described [69,70]. Briefly, two-month-old DBA/2J mice were intravitreally injected with 3 μl of PBS containing either 4.1 × 10^7^ vg AAV-eNdi1 (driven from the *CMV* promoter) or 4.1 × 10^7^ vg AAV-EGFP or PBS only. PhNRs and photopic a- and b-wave implicit times were recorded at 12 months of age, as described previously [69,70,116].

### 4.7. NADH Oxidation Rate Assay

NADH oxidation rates in retinas from one-year-old DBA/2J mice, intravitreally injected at two months of age with 4.1 × 10^7^ vg AAV-eNdi1 in one eye and an equal volume (3 μl) of PBS in the contralateral eye (n = 7), were compared. Additionally, ONs from similarly treated and untreated eyes (n = 4, where each sample represented 4 pooled ONs) were compared. Retinas and ONs were snap-frozen, and mitochondria were isolated as previously described [69]. NADH oxidation rate was determined by measuring the rate of decrease in absorbance at 340 nm, which occurs when NADH is oxidised to NAD+ and was normalised to total protein [72]. NADH oxidation rates on mitochondria were measured twice per sample, and duplicate readings were averaged as previously described [70,72]. In addition, following the addition of rotenone, 50 μM flavone was added to inhibit NDI1 protein activity to determine background levels of NADH oxidation rate. Total NADH oxidation rates, arising from both complex I and NDI1, were measured before the addition of the complex I inhibitor rotenone, while NADH oxidation rates from NDI1 alone (which is rotenone-insensitive) were measured after the addition of rotenone and before the addition of flavone.

### 4.8. Retinal Wholemounts and Immunohistochemistry

Mice were sacrificed at 1 year of age, and retinas were fixed in 4% paraformaldehyde. RGCs in whole retinas were stained with anti-BRN3A (Synaptic Systems, Goettingen, Germany) primary antibody and detected with Cy3-conjugated secondary antibody (Jackson ImmunoResearch Laboratories, West Grove, PA, USA), as previously described [70].

### 4.9. Fluorescence Microscopy

Microscopy was performed using an Olympus IX83 fluorescence microscope system (Mason Technology, Dublin, Ireland), SpectraX LED light source (Lumencor, Beaverton, OR, USA), and an Orca-Flash4.0 LT PLUS/sCMOS camera (Hamamatsu, Tsukuba City, Japan [109]). RGC quantification in retinal wholemounts was performed as described [70]. For the quantification of pLC cells, fluorescence intensity thresholds for CPN60 and DAPI signals were determined experimentally and set to differentiate between high and low label intensities. The same thresholds were used for all samples. The sum of the area of cells with a high-intensity threshold (CPN60) or the number of objects with high and high plus low signal intensities (DAPI) were determined using the Olympus CellSens software (v1.9).

### 4.10. Statistical Analysis

Statistical analysis was performed using GraphPad Prism (version 9.4; GraphPad Software, Boston, MA, USA). Student’s *t*-tests or paired Student’s *t*-tests were used to compare data between the two groups. ANOVA with Šídák’s multiple comparisons test was used to compare data between more than 2 groups. Differences were considered statistically significant at *p* < 0.05. Error bars represent standard deviations of the mean.

## Data Availability

The datasets used and/or analysed during the current study are available from the corresponding author upon reasonable request.
